# Hemotropic *Mycoplasma* spp. in Aquatic Mammals, Amazon Basin, Brazil

**DOI:** 10.3201/eid2812.220971

**Published:** 2022-12

**Authors:** Aricia Duarte-Benvenuto, Carlos Sacristán, Ana Carolina Ewbank, Irene Sacristán, Roberta Zamana-Ramblas, Waleska Gravena, Daniela M.D. Mello, Vera M. Ferreira da Silva, Miriam Marmontel, Vitor L. Carvalho, Juliana Marigo, José L. Catão-Dias

**Affiliations:** University of São Paulo, São Paulo, Brazil (A. Duarte-Benvenuto, C. Sacristán, A.C. Ewbank, R. Zamana-Ramblas, J. Marigo, J.L. Catão-Dias);; Centro de Investigación en Sanidad Animal, INIA-CSIC, Madrid, Spain (C. Sacristán, I. Sacristán);; Universidade Federal do Amazonas, Coari, Brazil (W. Gravena);; Instituto Nacional de Pesquisas da Amazônia, Manaus, Brazil (D.M.D. Mello, V.M. Ferreira da Silva);; Instituto de Desenvolvimento Sustentável Mamirauá, Tefé, Brazil (M. Marmontel);; Associação de Pesquisa e Preservação de Ecossistemas Aquáticos, Caucaia, Brazil (V.L. Carvalho)

**Keywords:** hemoplasmas, hemotropic Mycoplasma, bacteria, zoonoses, river dolphin, infectious diseases, molecular detection, aquatic mammals, Amazon Basin, Brazil

## Abstract

Hemotropic *Mycoplasma* spp. (hemoplasmas) are uncultivable bacteria that infect mammals, including humans. We detected a potentially novel hemoplasma species in blood samples from wild river dolphins in the Amazon River Basin, Brazil. Further investigation could determine pathogenicity and zoonotic potential of the detected hemoplasma.

Hemotropic *Mycoplasma* spp. (hemoplasmas) are uncultivable, cell-wall–deficient, pleomorphic bacteria that infect mammals, including humans (*1*). Although previously linked to anemia, starvation, and death, especially among immunosuppressed humans and animals (*2*,*3*), most hemoplasma species have subclinical manifestations (*1*). Hemoplasmas are thought to be host specific, but some reports suggest interspecies transmission and zoonotic potential (*3*–*5*). In aquatic mammals, hemoplasmas have only been reported in California sea lions (*Zalophus californianus*) (*6*).

Amazon river dolphins (*Inia geoffrensis*), Bolivian river dolphins (*I. boliviensis*), and Amazonian manatees (*Trichechus inunguis*) are endemic to the Amazon Basin. Both dolphin species have been classified as endangered, and *T. inunguis* manatees are classified as vulnerable (*7*). Infectious disease studies in these species are scarce. We used 16S rRNA PCR to detect and characterize hemoplasmas among aquatic mammals from the Amazon Basin Region, Brazil.

We analyzed blood samples of 50 wild dolphins, including 32 *I. geoffrensis* and 18 *I. boliviensis* dolphins live captured in scientific expeditions (*8*), during 2015 in the Guaporé and Negro Rivers; 2017 in the Tapajós River; and 2020 near Balbina hydroelectric dam (Table). We performed field hematology on wild dolphins and also analyzed blood samples collected during health assessments of 25 *T. inunguis* manatees under human care in Manaus in February 2022 (Appendix Tables 1, 2).

**Table:** Epidemiologic and molecular data of Hemotropic *Mycoplasma* spp. in aquatic mammals, Amazon Basin, Brazil*

Sample no.	Species	Age class/sex	Capture date	River	Hemoplasma detection	GenBank accession no.
1	*Inia geoffrensis*	Adult/F	2017 Oct 6	Tapajós	Y	ON711292
2	*I. geoffrensis*	Calf/M	2017 Oct 6	Tapajós	N	NA
3	*I. geoffrensis*	Adult/M	2017 Oct 7	Tapajós	N	NA
4	*I. geoffrensis*	Juvenile/M	2017 Oct 8	Tapajós	N	NA
5	*I. geoffrensis*	Juvenile/F	2017 Oct 10	Tapajós	Y	ON721292
6	*I. geoffrensis*	Adult/M	2017 Oct 10	Tapajós	Y	ON721292
7	*I. geoffrensis*	Adult/M	2017 Oct 10	Tapajós	Y	ON721292
8	*I. geoffrensis*	Adult/M	2017 Oct 11	Tapajós	Y	ON721300
9	*I. geoffrensis*	Adult/M	2017 Oct 11	Tapajós	Y	ON721302
10	*I. boliviensis*	Adult/M	2015 Feb 6	Guaporé	Y	ON721303
11	*I. boliviensis*	Calf/M	2015 Sept 22	Guaporé	Y	ON721296
12	*I. boliviensis*	Adult/M	2015 Sept 22	Guaporé	N	NA
13	*I. boliviensis*	Adult/F	2015 Sept 22	Guaporé	Y	ON721301
14	*I. boliviensis*	Juvenile/F	2015 Sept 22	Guaporé	N	NA
15	*I. boliviensis*	Juvenile/M	2015 Sept 22	Guaporé	N	NA
16	*I. boliviensis*	Adult/F	2015 Sept 23	Guaporé	N	NA
17	*I. boliviensis*	Calf/M	2015 Sept 23	Guaporé	N	NA
18	*I. boliviensis*	Adult/F	2015 Sept 23	Guaporé	Y	ON721296
19	*I. boliviensis*	Adult/M	2015 Sept 23	Guaporé	Y	ON721301
20	*I. boliviensis*	Adult/M	2015 Sept 24	Guaporé	Y	ON721297
21	*I. boliviensis*	Juvenile/M	2015 Sept 24	Guaporé	N	NA
22	*I. boliviensis*	Juvenile/M	2015 Sept 25	Guaporé	N	NA
23	*I. boliviensis*	Adult/M	2015 Sept 26	Guaporé	Y	ON121301
24	*I. boliviensis*	Adult/F	2015 Sept 27	Guaporé	Y	ON721296
25	*I. boliviensis*	Adult/M	2015 Sept 27	Guaporé	Y	ON121301
26	*I. boliviensis*	Adult/M	2015 Sept 27	Guaporé	Y	ON711298
27	*I. boliviensis*	Adult/M	2015 Sept 27	Guaporé	Y	ON721297
28	*I. geoffrensis*	Calf/M	2015	Negro	N	NA
29	*I. geoffrensis*	Adult/F	2020 Dec 2	Balbina	Y	ON721299
30	*I. geoffrensis*	Calf/F	2020 Dec 2	Balbina	N	NA
31	*I. geoffrensis*	Juvenile/M	2020 Dec 2	Balbina	N	NA
32	*I. geoffrensis*	Adult/M	2020 Dec 2	Balbina	Y	ON721299
33	*I. geoffrensis*	Adult/M	2020 Dec 2	Balbina	N	NA
34	*I. geoffrensis*	Juvenile/M	2020 Dec 3	Balbina	Y	ON721299
35	*I. geoffrensis*	Adult/M	2020 Dec 3	Balbina	N	NA
36	*I. geoffrensis*	Juvenile/M	2020 Dec 3	Balbina	Y	ON721299
37	*I. geoffrensis*	Juvenile/M	2020 Dec 4	Balbina	N	NA
38	*I. geoffrensis*	Juvenile/M	2020 Dec 4	Balbina	N	NA
39	*I. geoffrensis*	Juvenile/F	2020 Dec 4	Balbina	Y	ON721299
40	*I. geoffrensis*	Juvenile/M	2020 Dec 4	Balbina	Y	ON721299
41	*I. geoffrensis*	Adult/M	2020 Dec 4	Balbina	Y	ON721299
42	*I. geoffrensis*	Juvenile/M	2020 Dec 4	Balbina	Y	ON721295
43	*I. geoffrensis*	Juvenile/M	2020 Dec 5	Balbina	Y	ON721299
44	*I. geoffrensis*	Juvenile/M	2020 Dec 5	Balbina	Y	ON721299
45	*I. geoffrensis*	Adult/M	2020 Dec 5	Balbina	Y	ON721293
46	*I. geoffrensis*	Juvenile/M	2020 Dec 5	Balbina	Y	ON721293
47	*I. geoffrensis*	Juvenile/M	2020 Dec 5	Balbina	Y	ON721299
48	*I. geoffrensis*	Juvenile/M	2020 Dec 5	Balbina	Y	ON721299
49	*I. geoffrensis*	Adult/M	2020 Dec 6	Balbina	Y	ON721294
50	*I. geoffrensis*	Juvenile/M	2020 Dec 6	Balbina	N	NA

*Amazon river dolphins (*I. geoffrensis*) and Bolivian river dolphins (*I. boliviensis*) were live captured in scientific expeditions in Guaporé, Tapajós, and Negro rivers, and at the Balbina hydroelectric dam. NA, not applicable.

We extracted DNA by using the DNeasy Blood & Tissue Kit (QIAGEN, https://www.qiagen.com), following manufacturer instructions. We screened samples for *Mycoplasma* spp. by 16S rRNA PCR targeting a 384-bp fragment (*9*). We subjected positive samples to PCR targeting a 1,400-bp fragment of 16S rRNA (*10*) and confirmed amplicons by sequencing in both directions.

We used GraphPad Prism version 5 (GraphPad Software, https://www.graphpad.com) to compare prevalence among host species, sampling sites, sampling year, age, and sex, and hematological values in infected and noninfected animals; we considered p<0.05 statistically significant. We used the median joining method in PopART software (University of Otagao, https://www.popart.otago.ac.nz) to generate a nucleotide sequence type network. We assessed phylogeographic structure among species and sampling sites by using pairwise fixation index tests (FSTs) in Arlequin (http://cmpg.unibe.ch/software/arlequin3), determining level of significance with 1,000 permutations, and using the nearest-neighbor statistic (S_nn_) in DnaSP version 5 (Universitat de Barcelona, http://www.ub.edu/dnasp).

We detected *Mycoplasma* DNA in samples from 21 (65.6%, 95% CI 48.2%–83.0%) *I. geoffrensis* and 11 (61.1%, 95% CI 36.2%–86.1%) *I. boliviensis* dolphins. The percentage of *Inia* spp. dolphins testing hemoplasma-positive was higher than that reported for *Z. californianus* California sea lions (12.4%) (*6*). All manatees in our study tested PCR-negative for hemoplasma.

*Mycoplasma* nucleotide sequences from *Inia* spp. dolphins had <94.0% identity with the closest available sequence (GenBank accession no. CP003731), which was detected in alpacas (*Vicugna pacos*). We submitted 12 representative sequence types to GenBank (Table). Multilocus sequencing typing will be necessary to further characterize the *Mycoplasma* species we detected. 

Among animals sampled, adult dolphins had significantly higher hemoplasma prevalence than did calves (p = 0.0015). We saw no statistically significant differences among remaining variables, including the hematologic parameters between hemoplasma-positive and hemoplasma-negative dolphins; however, our sample size was small.

Network analyses differentiated the obtained nucleotide sequence types into 3 distinct groups: 1 comprises sequences of all *I. geoffrensis* dolphins samples from Balbina and Tapajós; the other 2, harbor sequences of all *I. boliviensis* dolphins samples from Guaporé, which are greatly divergent (Figure). Our analysis showed statistically significant differences among populations (S_nn_ = 1.0, p = 0.0001; FST = 0.48, p = 0.003), confirming a geographic genetic structure. Haplotype diversity (Hd), average number of nucleotide differences (K), and nucleotide diversity (π) were higher among animals from Guaporé compared with the other 2 sites. For Guaporé, Hd was 0.82, K 43.6, and π 0.03; for Tapajós, Hd was 0.4, K 0.4, and π 0.0003; and for Balbina, Hd was 0.44, K 0.71, and π  0.0005. We also noted that *Mycoplasma* among host species shared genetic structure that differed between the 2 *Inia* species (Snn = 1.0, p = 0.0001; FST = 0.43, p = 0.000). The genetic structure difference between the species and sites likely reflects geographic separation of the studied populations (Appendix Figure 1). However, geographic separation does not explain the hemoplasma divergence between the 2 sequence types collected from *I. boliviensis* dolphins. All retrieved sequences clustered together and with other hemoplasma sequences of unknown pathogenicity (Appendix Figure 2).

**Figure:**
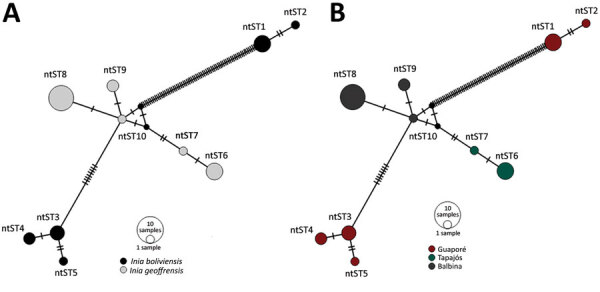
ntST network analyses of hemotropic *Mycoplasma* spp. (hemoplasmas) from aquatic mammals, Amazon Basin, Brazil. We noted hemoplasmas divergence between 2 dolphin species (A) and sampling sites (B). The analysis differentiated the retrieved hemoplasmas nucleotide sequence types in 3 distinct groups: 1 group comprised all sequences obtained from Amazon river dolphins (*Inia geoffrensis*) from the Balbina Dam and Tapajós River; the other 2 harbored all sequences from Bolivian river dolphins (*I. boliviensis*) from the Guaporé River. ntST, nucleotide sequence type.

Our findings indicate that aquatic mammals can be infected by hemoplasmas, but epidemiology remains unknown. In terrestrial mammals, hematophagous vectors are the main proposed transmission route (*1*). *T. inunguis* manatees in our study tested hemoplasma-negative despite being housed in tanks close to the forest without vector protection. This finding suggests food could be a transmission route among aquatic mammals because river dolphins are piscivorous and manatees are herbivorous. Also, 5 female dolphins captured with calves tested positive, but the calves tested negative, which might exclude vertical transmission. Endoparasitism or direct contact are other possible transmission routes.

In conclusion, we detected hemoplasmas in *I. geoffrensis* and *I. boliviensis* river dolphins. Pathogenicity and zoonotic potential require further investigation, but the high hemoplasma prevalence in adult mammals and detection among animals over several years suggest hemoplasma endemicity in these dolphin populations. 

Appendix Additional information on hemotropic *Mycoplasma* spp. in aquatic mammals, Amazon Basin, Brazil.
