# Supplementary material for: Hemotropic Mycoplasma spp. in Aquatic Mammals, Amazon Basin, Brazil
Source: Emerg Infect Dis. 2022 Dec;28(12):2556–9. doi: 10.3201/eid2812.220971 (PMC9707567; doi:10.3201/eid2812.220971)
Supplement: Appendix — Additional information on hemotropic Mycoplasma spp. in aquatic mammals, Amazon Basin, Brazil. [file 22-0971-Techapp-s1.pdf]

Article DOI: https://doi.org/10.3201/eid2812.220971

Hemotropic *Mycoplasma* spp. in Aquatic Mammals, Amazon Basin, Brazil

Appendix

Appendix Table 1. Hematology of Amazon river dolphins (*Inia geoffrensis*) captured in Balbina in 2020 in a study of Hemotropic mycoplasma in Aquatic Mammals, Amazon Basin, Brazil*

				Cells $\times 10^9$/L							
Sample no.	RBC, $\times 10^{12}$/L	Hct, L/L	MVC, fL	Platelets	Leuk	Monocyte (%)	Neutrophil (%)	Band (%)	Lymphocyte (%)	Eosinophil (%)	Baso (%)
29	3.44	0.40	107.24	ND	8.45	0.34 (4)	4.14 (49)	0	3.21 (38)	0.76 (9)	0
30	3.67	0.36	104.65	ND	10.7	0.74 (7)	7.28 (68)	0	2.46 (23)	0.21 (2)	0
31	3.18	0.38	103.54	ND	8.9	0.27 (3)	4.72 (53)	0	2.85 (32)	1.07 (12)	0
32	3.99	0.36	113.21	ND	9.85	0.30 (3)	6.30 (64)	0	1.08 (11)	2.17 (22)	0
33	3.11	0.38	95.24	ND	13.85	0.55 (4)	10.25 (74)	0	1.39 (10)	1.66 (12)	0
34	3.31	0.38	122.19	300	10.65	0.11 (1)	6.60 (62)	0.11 (1)	1.92 (18)	1.92 (18)	0
35	2.88	0.38	114.80	352	7.35	0.15 (2)	4.19 (57)	0	1.25 (17)	1.76 (24)	0
36	4.52	0.38	135.42	280	10	0.30 (3)	7.60 (76)	0.1 (1)	1.60 (16)	0.40 (4)	0
37	3.47	0.39	88.50	190	10.05	0.20 (2)	6.43 (64)	0	1.91 (19)	1.51 (15)	0
38	3.49	0.40	109.51	60	6.5	0.13 (2)	4.29 (66)	0.07 (1)	1.17 (18)	0.91 (14)	0
39	3.16	0.38	108.88	256	9.5	0.38 (4)	6.75 (71)	0	1.81 (19)	0.57 (6)	0
40	3.68	0.38	129.75	190	7.75	0.31 (4)	3.49 (45)	0	2.33 (30)	1.63 (21)	0
41	3.02	0.41	95.11	110	10.6	0.32 (3)	7.53 (71)	0	1.70 (16)	1.06 (10)	0
42	2.78	0.35	139.07	298	7.1	0.14 (2)	4.69 (66)	0	0.57 (8)	1.70 (24)	0
43	3.22	0.42	136.69	292	8.85	0.35 (4)	5.31 (60)	0	1.68 (19)	1.50 (17)	0
44	3.64	0.38	111.80	154	5.8	0.12 (2)	3.48 (60)	0	1.22 (21)	0.99 (17)	0
45	3.64	0.36	104.40	194	7.8	0.39 (5)	4.29 (55)	0	1.95 (25)	1.17 (15)	0
46	3.3	0.38	104.40	336	9.5	0.38 (4)	5.80 (61)	0	0.86 (9)	2.47 (26)	0
47	4.47	0.40	121.21	234	12.35	0.12 (1)	6.92 (56)	0	2.10 (17)	3.21 (26)	0
48	3.37	0.41	91.72	166	14.5	0.58 (9)	10.44 (72)	0	1.31 (9)	2.18 (15)	0
49	3.13	0.40	118.69	346	6.85	0.41 (18)	4.11 (60)	0	1.23 (18)	1.10 (16)	0
50	3.44	0.35	111.82	150	8.95	0.36 (11)	6.62 (74)	0	0.98 (11)	0.98 (74)	0
Ref†	3.77–<br>4.01	0.39–<br>0.40	101–107	277–317	15.8–<br>17.4	0.35–<br>1.04<br>2.47–<br>3.24	7.64–8.91<br>46.16–<br>49.65	0.14–<br>0.24<br>0.83–<br>1.30	5.68–6.91<br>33.41–<br>36.75	2.04 –<br>2.79<br>11.86–<br>14.27	ND

*Hematology performed according to conventional techniques for manual cell counting in mammals. Baso, basophil; HCT, hematocrit; Leuk, leukocyte count; MVC, mean corpuscular volume; RBC, red blood cell count; Ref, reference range.
†Mello DMDd, da Silva VMF. Hematologic profile of Amazon river dolphins *Inia geoffrensis* and its variation during acute capture stress. PLoS ONE. 2019;14(12):e0226955.

Appendix Table 2. Epidemiologic data and outcome of Amazonian manatees (*Trichechus inunguis*) under human care in Manaus (Amazonas state, Brazil) in a study of Hemotropic mycoplasma in Aquatic Mammals, Amazon Basin, Brazil*

Sample no.	Age class	Sex	Year of rescue	Outcome
1	Calf	F	2020	Under rehabilitation
2	Juvenile	F	2019	Under rehabilitation
3	Juvenile	F	2019	Under rehabilitation
4	Juvenile	M	2019	Under rehabilitation
5	Adult	F	1974	Under rehabilitation
6	Juvenile	F	2019	Under rehabilitation
7	Juvenile	M	2018	Under rehabilitation
8	Juvenile	M	2019	Under rehabilitation
9	Adult	F	2005	Under rehabilitation
10	Adult	F	2002	Permanent resident
11	Juvenile	M	2016	Under rehabilitation
12	Calf	M	2019	Under rehabilitation
13	Juvenile	M	2019	Under rehabilitation
14	Calf	M	2019	Under rehabilitation
15	Adult	F	1984	Under rehabilitation
16	Juvenile	F	2019	Under rehabilitation
17	Adult	F	1984	Under rehabilitation
18	Calf	M	2020	Under rehabilitation
19	Calf	F	2020	Under rehabilitation
20	Juvenile	M	2019	Under rehabilitation
21	Adult	M	2021	Under rehabilitation
22	Adult	F	2013	Under rehabilitation
23	Juvenile	M	2020	Under rehabilitation
24	Juvenile	F	2019	Under rehabilitation
25	Juvenile	M	2018	Under rehabilitation
26	Adult	M	2007	Under rehabilitation

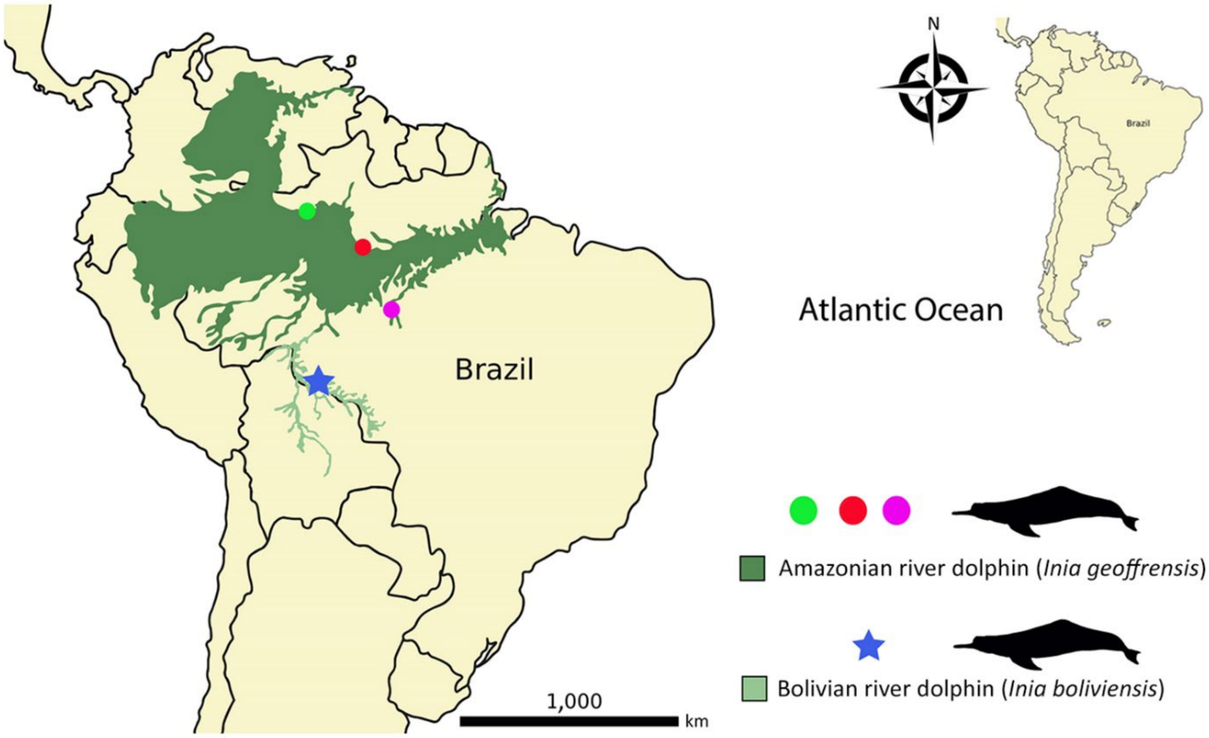

Appendix Figure 1. Map of sampling sites in a study of Hemotropic mycoplasma in aquatic mammals, Amazon Basin, Brazil. Inset shows the location of the study area in South America. Dark green area indicates range of Amazon river dolphins (*Inia geoffrensis*), which we captured and sampled in 3 locations: Negro River (green dot), Balbina Dam (red dot), and Tapajós River (pink dot). Light green area indicates range of Bolivian river dolphins (*I. boliviensis*), which we captured and sampled from the Guaporé River (blue star).

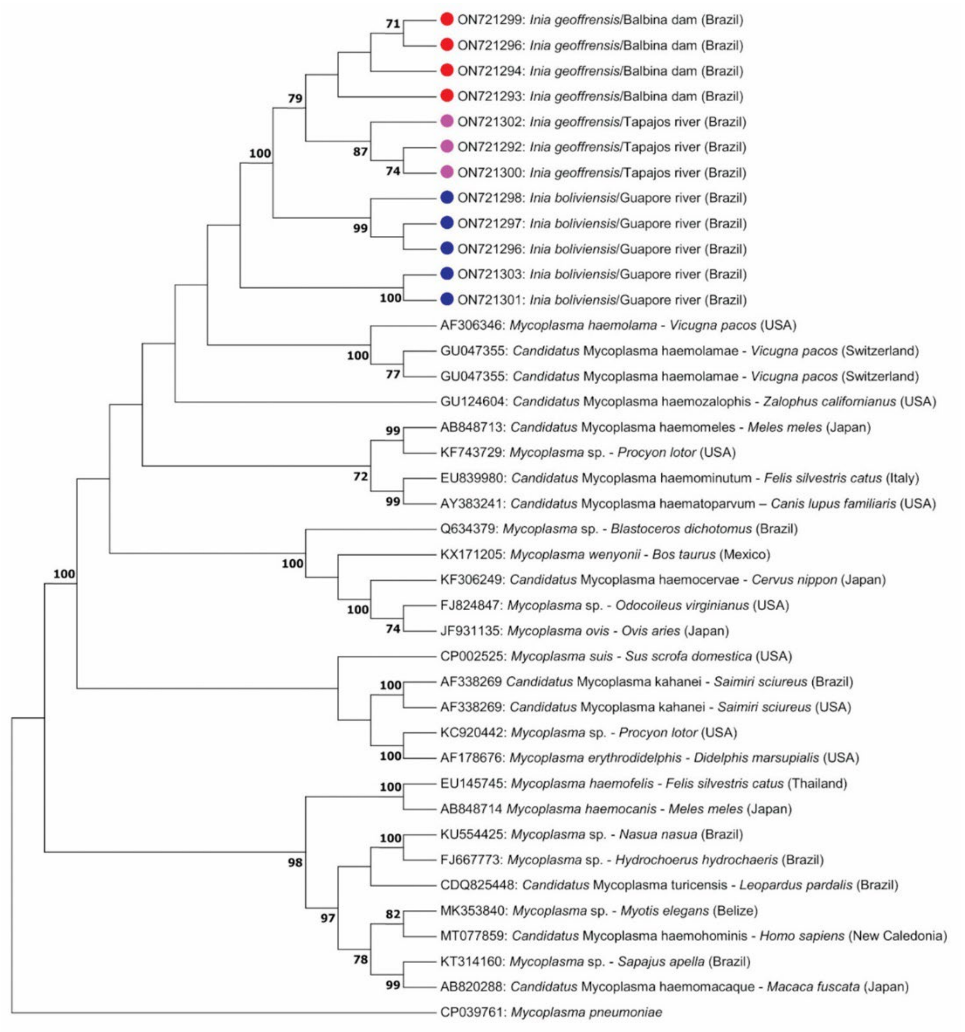

Appendix Figure 2. Phylogenetic tree of Hemotropic mycoplasma in aquatic mammals, Amazon Basin, Brazil. We used general time-reversible plus inversion plus gamma distribution to create a maximum-likelihood phylogram of a 1,200-bp fragment of mycoplasma nucleotide sequences obtained from aquatic mammals in this study and other hemotropic mycoplasma sequences retrieved from GenBank. We selected *Mycoplasma pneumoniae* as an outgroup. Red dots indicate sequences obtained from dolphins near Balbina Dam; pink dots from dolphins in Tapajós River; and blue dot is from dolphins in Guaporé River.
